# Optimising pain management in intensive care setting: Insights from a realist evaluation

**DOI:** 10.1016/j.ijnsa.2026.100515

**Published:** 2026-02-23

**Authors:** Samira Hamadeh, Loretta Garvey

**Affiliations:** Institute of Health and Wellbeing, Federation University Australia, Australia

**Keywords:** Implementation, Intensive care, Interventions, Nursing, pain management, Social system, Stakeholders

## Abstract

**Background:**

Inconsistent pain management in intensive care settings contributes to poor patient outcomes and prolonged hospital stays. Implementing evidence-based pain management strategies is critical to mitigating complications, such as oversedation and excessive analgesia. The process is inherently complex, requiring an exploration of contextual factors that enable implementation, the mechanisms triggered within these contexts, and strategies to enhance stakeholder engagement.

**Objective:**

In this discussion paper, we examined sociocultural, organisational, professional, and individual factors influencing the implementation of pain management interventions in intensive care settings. We considered their implications for diverse stakeholders and have offered evidence-informed recommendations to strengthen implementation.

**Information Source:**

In this discussion we synthesise findings from a theory-driven, three-phase realist evaluation that examined and refined program theories related to successful implementation of pain management interventions. Phase one involved a scoping review of the literature and a survey with open-ended questions to elicit initial program theories. Phases two and three comprised a rapid realist review followed by stakeholder interviews to iteratively refine these theories.

**Results:**

Interventions succeed or fail not solely based on design but within complex contexts where knowledge, power, and professional identity intersect. Across micro, meso, and macro levels of care, mechanisms such as empowerment, moral distress, and institutional trust influenced intensive care nurses’ willingness and capacity to adopt interventions. Biomedical hierarchies may marginalise nursing knowledge, while interprofessional hierarchies and organisational cultures can either constrain or enable meaningful change.

**Conclusion:**

We have advanced critical nursing scholarship by conceptualising pain management not merely as a clinical intervention but as a socially embedded practice. We have offered implications for educators, policymakers, nurse leaders, and practitioners to promote equitable, context-sensitive strategies for implementing pain management interventions, thereby possibly enhancing clinical practice and improving patient outcomes.

**What is already known**

• Pain management practices lack consistency, which affects patient outcomes.
• Implementing pain management interventions can influence the social dynamics within a healthcare facility, thereby shaping the motivations, capabilities, and opportunities available to stakeholders.
• Service delivery contexts are inherently dynamic, and the successful implementation of interventions depends on the presence of supportive contextual conditions.

Alt-text: Unlabelled box dummy alt text

**What this paper adds**

• Actions at the micro, meso and macro level of the health facility’s social system may be paramount to enhance adherence to and sustainability of interventions.
• Actions that foster multi-level system engagement may trigger increased confidence and cultivate empowerment, self-efficacy, reflective motivation, trust, awareness, and autonomy.
• Such actions may enhance organisational capacity, mitigate frustration stemming from inconsistent practices and strengthen accountability and ownership, contributing to improved implementation outcomes.

Alt-text: Unlabelled box dummy alt text

## 1 Introduction

Pain is an unpleasant feeling that demands prompt assessment and diligent management because it can aggravate a critically-ill patient’s condition, resulting in haemodynamic instability, respiratory compromise, and immunosuppression (Devlin et al., 2018). Pain management, particularly in the intensive care unit, entails a substantial level of competence and a patient-centred approach to reduce suffering, avert over-sedation, and counteract undesirable consequences, including prolonged hospital stays, extended time attached to mechanical ventilators and increased mortality rates (Soares Pinheiro et al., 2020). The ability of patients in intensive care units to verbally communicate pain levels is impeded by the constraints of intubation and sedation (Herr et al., 2019). Hence, pain management is challenging, influenced by patient ailment and treatment modalities used to sustain and restore life (Nordness et al., 2021). In the wider literature, numerous valid and reliable tools to observe changes in patient behaviour and detect pain were cited (Gélinas et al., 2006; Klein et al., 2010; Latorre Marco et al., 2011; Payen et al., 2001). Additionally, guidelines for pain management in intensive care settings emphasised regular and accurate pain assessment, adopting an analgesia-first approach before initiating sedation, and implementing targeted pharmacologic and non-pharmacologic strategies to optimise patient outcomes and minimise the adverse effects of sedation(Devlin et al., 2018; Nordness et al., 2021). However, pain across intensive care settings is still not managed consistently and effectively, even where guidelines and validated tools are recommended in practice (Hamadeh et al., 2024b; Kerbage et al., 2021). While interventions targeting pain, sedation, delirium, and health-related quality of life are increasingly adopted, strategic approaches are essential to ensure effective implementation, foster stakeholder engagement, and support long-term sustainability (Sosnowski et al., 2023). Policy makers wrongly assume that health organisations are ready to engage in the process of change and mistakenly believe stakeholders possess necessary adaptive capacity to do so. Given the dynamic nature of intensive care settings, effective implementation of pain management interventions requires understanding the mechanisms that facilitate change, engaging stakeholders, and overcoming entrenched practices and beliefs.

It is even more critical to take this a step further and discover the contextual features enabling adherence to, and sustainability of, pain management interventions. Whether these features are conducive to learning, innovation and growth can affect readiness to induce practice change.

## 2 Background

Pain management presents significant complexity due to an interplay of multiple factors that shape pain experience. Understanding how, why, and under what conditions interventions targeting this critical aspect of nursing care can be effectively implemented is essential. Critical realism, operationalised through realist evaluation, is increasingly used to explain why complex interventions produce varied outcomes (De Weger et al., 2020). By examining mechanisms triggered in specific contexts, realist evaluation makes causal processes explicit (Westhorp et al., 2011). Rooted in Bhaskar’s work, critical realism posits that knowledge of reality is socially constructed yet anchored in an objective world that enables theory testing (Archer et al., 2013; Bhaskar, 2013). Complete understanding is unattainable because knowledge is shaped by historically-contingent discourses and power structures that define what can be known (Foucault & Nazzaro, 1972). Realist philosophy emphasises that observational evidence alone cannot establish causality; mechanisms, underlying causal forces within entities, processes, or social structures are activated in particular contexts to generate outcomes (Wong et al., 2013). Consequently, similar interventions may trigger different mechanisms even in the same setting, and programs implemented across diverse contexts often yield distinct results (Pawson & Tilley, 1997; Wong et al., 2013). For interventions to succeed, contextual features influencing stakeholder reasoning must align with mechanisms that drive adherence and produce intended outcomes (Jagosh et al., 2022; Mukumbang, 2020).

Comprehensive inquiries go beyond induction and deduction, which alone risk producing ontologically flat explanations (Bhaskar, 2010; Jagosh, 2020). Induction describes what happens without explaining why, while deduction predicts what should happen without empirical grounding (Bhaskar, 2010; Jagosh, 2020). Retroduction, the overarching logic in realist evaluation, integrates induction, deduction, and abduction to uncover causal mechanisms underlying observed events (Mukumbang et al., 2021; Sayer, 1992).

In this discussion, we have drawn on a three-phase realist evaluation to explore how implementation of pain management interventions can be optimised. Multiple evidence sources, informed by exploratory quantitative and qualitative approaches, were consulted to construct the realist-informed program theory (Mukumbang, 2023).

**Fig. 1:**
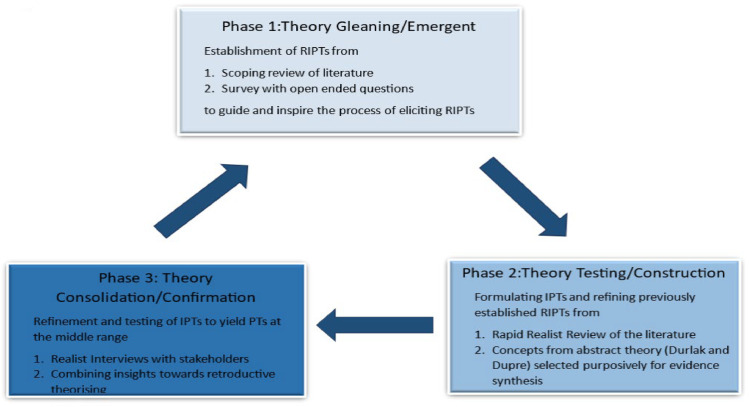
Phases and methods employed to refine and derive program theories.

Fig. 1 provides an overview of the realist evaluation process, outlining the phases and corresponding methods that informed the development of this paper

*Note –* RIPTs refer to rough initial program theory; IPT refer to initial program theory; PTs refer to program theory at the middle range

As presented in Fig. 1, the realist evaluation encompassed the following phases.

1.Theory Gleaning Phase, informed by a scoping review of the literature and a survey with free-text responses (Hamadeh et al., 2024a; Kerbage et al., 2021). From a realist perspective, the scoping review explored how pain management practices are shaped by attitudes, values, beliefs, and guidelines. Identified gaps in the literature provided the impetus for further research, prompting consideration of existing theoretical frameworks and their utility in interpreting current descriptions of pain management. Survey responses generated preliminary theories, which were refined and tested to assess their value in identifying factors that optimise pain management implementation and explain their effects (Duddy & Wong, 2018). These theories were framed as Context-Mechanism-Outcome configurations for testing and refinement in subsequent phases.
2. Theory Testing/Construction Phase, in which rough initial program theories were refined by consulting evidence from the literature/extant theories(Hamadeh et al., 2024b). We employed a rapid realist review to synthesise existing evidence to build and refine theory, track earlier program theories, and generate testable hypotheses for subsequent evaluation(Pawson et al., 2005; Pawson & Tilley, 2004). We examined mechanisms and contextual factors influencing acceptance/resistance to implementation, focusing on individual, interpersonal, infrastructural, and institutional conditions.
3. Theory Consolidation Phase, in which program theories were further refined and tested to achieve abstraction by collating insights from nurses, nurse educators, and nurse team leaders(Hamadeh et al., 2024d). To deepen understanding of how pain management interventions can be implemented effectively in intensive care units, we conducted realist interviews to capture stakeholder perspectives, refine initial program theories, and delineate what works, for whom, under which circumstances, why, and to what extent (Hewitt et al., 2014). A summary of the refined program theories is provided in Table 1 for reference.

**Table 1:** Refined program theories derived from the three-phase realist evaluation study.

Refined Program Theory	Level of the Social System
Health organisations provide training and education, which makes nurses empowered, boosts their confidence, and makes them feel valued.	MACRO
Health organisations strive to update the evidence for practice, use valid and reliable interventions, and build awareness on their benefits to reassure nurses; this triggers confidence and self-worth.	
Health organisations institute quality assurance structures and provide constructive feedback, which motivates nurses and activates a sense of accountability and ownership of behaviour.	
Health organisations develop stakeholders’ interrelationships and shared mental models through effective communication, which triggers motivation and fosters a culture of equality and trust, and enable team members to interpret situations similarly, anticipate each other’s needs, coordinate implicitly, and collaborate more effectively	
Health organisations provide resources, adjust workloads to minimise nurses’ frustration, which makes them feel supported, reassured, and less pressured.	
Health organisations improve infrastructure, overcome barriers to implementation of interventions, and reward performance, which activates a sense of satisfaction and reassurance and builds capacity	
Team leaders strive to develop stakeholder inter/intrarelationships and professional identity,which cultivates a sense of empowerment and abolishes apprehension and powerlessness resulting from interdisciplinary hierarchies.	MESO
Team leaders provide access to opportunity, validate the nurse’s role and support autonomy in decision-making to build enthusiasm for knowledge acquisition and create incentives for driving improvements.	
Team leaders change priorities and models of care, which triggers enthusiasm to buy into interventions and reconciles team disagreements affecting intrinsic motivation.	
Nurses understand ethical responsibilities and strive to implement interventions driven by an obligation to reduce the risk of harm and duty of care; nurses actively pursue knowledge to build their capability activating reflective motivation.	MICRO
Nurses demonstrate flexibility and acceptance of newly-implemented interventions and engage in reflective practices, which triggers a sense of responsibility to establish effective communication within the interprofessional team.	
Nurses assert self-determination and overcome doubts, which boosts autonomy driving forth implementation.

Drawing on the preceding realist evaluation, we discuss sociocultural, organisational, professional, and individual factors shaping the implementation of pain management interventions, outlining implications for stakeholders and offering recommendations to strengthen uptake. We critically explore contextual and professional dynamics, emphasising the relational and epistemic work of nurses within technocratic healthcare systems.

## 3 Overview of contextual influences

The macro (organisational/professional), meso (team-based), and micro (individual) layers of the social system are deeply interconnected and do not function independently. Each level influences and reinforces the others; for instance, organisational culture at the macro level shapes team dynamics at the meso level, which subsequently affects individual behaviours and attitudes at the micro level. Attempting to isolate these layers would oversimplify their complexity and conflict with the realist approach, which emphasises the interdependence of context, mechanism, and outcome.

### 3.1 At the macro level

#### 3.1.1 Building capacity through education and organisational support

Change that warrants implementation of interventions must align with the dynamic demands of patient care and the unpredictability of critical illness. Health administrations and policymakers play a pivotal role in supporting stakeholders, providing resources, and fostering capacity building to achieve meaningful outcomes. Pain management practices often diverge from published guidelines (Elliott et al., 2013; Kerbage et al., 2021), largely due to inadequate knowledge, limited training in behavioural observation tools, and absence of standardised protocols (Hamadeh et al., 2024a). While some hospital offer nurses pain management training, it remains a neglected priority in others, with an implicit expectation that nurses possess these competencies, despite insufficient evidence that they do (Hamadeh et al., 2024a). Lack of training impedes knowledge translation into practice (Wysong, 2014). Healthcare organisations can work together with clinical teams to prioritise evidence-based education, support the use of valid assessment tools, and integrate pain assessment as a shared nursing competency.

#### 3.1.2 Advancing nurse autonomy in critical care

Professional autonomy is central to nursing practice, yet decision-making is dominated by a reductionist medical model and hierarchical structures favouring intensivist-led teams (Weled et al., 2015). Licensed providers with prescriptive authority, such as physicians, primarily determine pharmacologic choices, dosing, and sedation protocols, while nurses often rely on pattern recognition and physician orders, limiting nurses’ scope. Cultural change is needed to empower nurses, validate their role, and promote strength-based approaches. Autonomy is closely linked to access to education and collaborative interprofessional relationships that foster mutual respect and shared decision-making (Georgiou et al., 2017; Pursio et al., 2021). Organisational cultures that encourage flexibility, capacity building, and recognition of staff contributions drive positive outcomes and sustain practice change (Kowal, 2010; Latimer et al., 2010). Transformational leadership further supports environments of shared responsibility, trust, and adaptive practices, enabling sustainable implementation (Durlak, 2017).

#### 3.1.3 Addressing sociocultural challenges in practice change

Resistance to change often stems from behavioural, cognitive, and affective factors (Mareš, 2018). Nurses may resist interventions due to overconfidence in existing skills or fear of disrupting established routines (Erwin & Garman, 2010; Hamadeh et al., 2024c). Addressing these concerns requires clear communication of benefits and consideration of sociocultural implications to prevent implementation barriers (Tsang et al., 2019).

#### 3.1.4 Fostering engagement through incentives, trust, and collaborative systems

Aligning incentives through recognition and reward systems is critical for fostering staff engagement with new interventions. Short-term incentives should align with the organisation’s mission and values to sustain buy-in and avoid internal conflict, promoting a unified approach to change (George & Massey, 2020). Building trust between nurse managers and teams encourages open communication and collaborative problem-solving (Dorgham & Al-Mahmoud, 2013). Supportive structures that empower frontline staff and ensure all voices are heard are essential for integrating interventions into practice (Hamadeh et al., 2024c).

### 3.2 At the meso level

#### 3.2.1 Empowering teams through collaborative leadership

Collaborative leadership is pivotal in enabling clinicians to make informed decisions and assume personal ownership of pain management interventions. The behaviours modelled by team leaders shape organisational practice and service delivery. Indeed, inadequate team alignment has been identified as a root cause of implementation challenges (Khan et al., 2020). Cultural beliefs and priorities within healthcare teams guide stakeholder choices, drive commitment, and foster alignment in patient care (Hamadeh et al., 2024c). Unit cultures that value lifelong learning, enable open communication, and demonstrate supportive attitudes enhance readiness for change (Hamadeh et al., 2024c). When team leaders build awareness of the imperative for performance-enhancing interventions, cultivate shared decision-making at the unit level, ensure appropriate resourcing, and institute supportive infrastructure, teams are more likely to endorse implementation (Durlak & DuPre, 2008). These contextual features promote empowerment and collective ownership of the intervention (Hamadeh et al., 2024b). Empowerment is associated with increased assurance, satisfaction, and self-worth. Team leaders who empower stakeholders can facilitate the abandonment of entrenched beliefs and the adoption of constructive behaviours, thereby improving implementation outcomes (Kanter, 1993).

#### 3.2.2 Shared decision-making and mental models: foundations for interprofessional collaboration

Shared interprofessional decision-making strengthens team belonging and solidarity, while building trust and professional equality. Team leaders who nurture shared mental models enable teams to thrive amid change and to embrace intervention implementation (Kowal, 2010). Shared mental models are cognitive structures that enable team members to interpret situations, similarly, anticipate each other’s needs, coordinate implicitly, and collaborate more effectively. When teams share these models, they require less explicit communication and are better able to adapt in dynamic or high-pressure environments (Mathieu et al., 2000). These contextual features directly influence motivation and opportunity, thereby supporting behaviour change (Michie et al., 2011).

#### 3.2.3 Mitigating stressors and empowering nurses through structured support mechanisms and access to opportunity

Provision of targeted supports enhances individual’s capacity to learn about the intervention and mitigates stressors associated with workload, time constraints, and the demands of critical illness (Hamadeh et al., 2024a). Training and technical assistance build confidence and skills, increasing autonomy and the likelihood of engagement (Durlak, 2017). Educational investment signals value and recognition, nurturing self-worth and motivation to change practice (Hamadeh et al., 2024d). The involvement of intervention champions (e.g., clinical nurse specialists) provides guidance, bolsters confidence, and reduces uncertainty throughout the implementation process (Hamadeh et al., 2024d). Junior staff, who may lack the experience to translate theoretical knowledge safely into practice, benefit from accessible resource persons to support decision-making (Massey et al., 2015).

#### 3.2.4 Fostering accountability and transparency through inclusive implementation practices

Endorsement and implementation of evidence-based interventions that align with stakeholder expectations are closely linked to positive implementation outcomes (Durlak & DuPre, 2008). Empowering stakeholders to provide feedback on relevance, quality, appropriateness, and effectiveness ensures their voices inform decisions (Hamadeh et al., 2024b). Creating opportunities for open dialogue about challenges, errors, and frustrations fosters accountability and transparency, while also generating practical solutions to emerging concerns (Hamadeh et al., 2024d). Consequently, sustained stakeholder engagement, across all stages from initial conception and development through to implementation and evaluation, is essential for successful and enduring adoption of interventions (Hamadeh et al., 2024d).

#### 3.2.5 Fostering belonging and engagement through recognition and cultural alignment

At the unit level, recognition of contributions strengthens belonging and can trigger intrinsic motivation, a precursor to self-determined action and behaviour change (Messineoet al., 2019; Michie et al., 2011). Feelings of empowerment among nurses are grounded in being valued as contributors to the implementation process; conversely, disenfranchisement and disengagement undermine intervention buy-in (Hamadeh et al., 2024c). Unit culture influences stakeholders’ experience: clear expectations and explicit recognition of individual effort are essential (Hamadeh et al., 2024d). A unit leadership that recognises individual effort, stimulates motivation, and rewards excellence is more likely to unify teams and achieve implementation goals, enhancing perceived value, morale, job satisfaction, and productivity (Kohnen et al., 2024). Across implementation stages, supportive leadership practices, such as listening, engaging, resolving conflict, mitigating barriers, flattening interprofessional hierarchies, and ensuring appropriate dissemination, are critical (Hamadeh et al., 2024c). Implementation success is also contingent on cultivating a supportive culture that sustains professional identity. Nurses develop professional identity by integrating personal values with professional and societal norms (Crigger & Godfrey, 2014) and frequently emulate prototypical characteristics distilled from leadership behaviours (Willetts & Clarke, 2014).

#### 3.2.6 Instituting performance evaluation and upholding accountability for continuous improvement in clinical practice

Stakeholders’ ethical commitments to avoid harm heighten the value placed on evidence-based interventions. Quality assurance mechanisms, including systematic evaluation of practice, structured feedback, adequate resourcing, and scheduled reviews to assess suitability, effectiveness, and efficiency, are necessary to sustain implementation (Manghani, 2011). At the unit level, regular performance evaluation encourages reflection, ownership, accountability, and promotes professional growth (Hamadeh et al., 2024d).

Following the analysis of meso-level contextual factors, we now turn the discussion to micro-level determinants within the organisation’s social system.

### 3.3 At the micro level

#### 3.3.1 Shared decision-making and infrastructure as catalysts for nursing autonomy in pain care

When nurses recognise the value of a pain management intervention within a supportive environment, characterised by shared decision-making, adequate resources, and robust infrastructure, they are more likely to engage in its implementation and sustain its use. This sense of agency fosters professional autonomy, enabling nurses to tailor intervention delivery to local needs and cultural expectations (Hamadeh et al., 2024c). In such contexts, nurses develop community ownership of the intervention and trust in the organisation (Hamadeh et al., 2024d). Collaboration among key stakeholders in a positive work climate, open to change and equipped with capacity-building resources, facilitates successful implementation (Durlak & DuPre, 2008).

#### 3.3.2 Feeling empowered triggers nurses’ engagement

Trust in leadership and colleagues is associated with organisational citizenship behaviours, such as conscientiousness, civic virtue, courtesy, and altruism (Altuntas & Baykal, 2010). Being open to constructive feedback and interprofessional collaboration encourages original thinking and active participation in decision-making (Kanter, 1993). Feeling empowered and valued motivates nurses to achieve goals and empower others, improving practice and organisational outcomes (Kanter, 1993). Psychological empowerment, defined as the perception of meaning, competence, self-determination, and impact, correlates with lower burnout, stronger organisational commitment, and greater job satisfaction (Ouyang et al., 2015; Oyeleye et al., 2013; Spreitzer, 1995). Empowerment is a key mechanism driving implementation outcomes; nurses are more likely to accept a pain management intervention when it enhances competence, supports self-determined choice, and positively influences patient and organisational outcomes (Hamadeh et al., 2024b). Empowering nurses to contribute to policy development positions them as proactive change agents rather than passive recipients of directives (Hamadeh et al., 2024d).

#### 3.3.3 Prevailing over power dynamics and autonomy as drivers for implementation

Contexts that promote balanced power dynamics and reduce hierarchical barriers motivate nurses to uphold a positive professional image and prioritise best-practice care (Laporta et al., 2005). Conversely, settings where physicians dominate decision-making and nurses’ clinical judgment is undervalued hinder active participation and foster feelings of subservience. Promoting autonomy and providing opportunities for professional growth counteract stagnation and resistance to change (Laporta et al., 2005). Empowering nurses to contribute to policy development positions them as proactive change agents rather than passive recipients of directives (Hamadeh et al., 2024d). Strong infrastructure, including clinical educators, advanced practice nurses, and support staff, is essential for fostering collaboration during implementation (Hamadeh et al., 2024d). Furthermore, raising awareness of ethical and moral obligations related to pain management activates self-determined engagement (Messineoet al., 2019).

#### 3.3.4 Overcoming negative perceptions and engaging in reflective practices

Negative attitudes and misconceptions about interventions impede intervention buy-in, consistent with broader evidence (Alotni et al., 2023; Deldar et al., 2018; Ista et al., 2013; Raurell-Torredà et al., 2019). Comprehensive pain management programs enhance awareness and positively influence attitudes (Sedighie et al., 2020). Encouraging reflective practice helps nurses avoid assumptions and routine-driven behaviours (Reid, 1993). Senior nurses may resist change due to limited knowledge or appreciation of interventions, particularly when evidence of improved patient outcomes is perceived as inconclusive (Hamadeh et al., 2024d).

#### 3.3.5 Asserting professional identity

Professional identity, which denotes nurses’ self-concept of competence and role, intersects with their ability to meet quality standards (Björkström et al., 2008). Intensive care nurses’ perception of their professional position within the social system influences acceptance of change, especially when change introduces dissonance between expectations and experiences (MacIntosh, 2003).

Nurses’ ability to present a strong image of competence is integral to their professional identity and credibility within the healthcare team. Demonstrating clinical expertise not only reinforces trust among colleagues and patients but also positions nurses as equal contributors in decision-making processes. This projection of competence is closely linked to self-determined choices, which reflect autonomy and confidence in professional judgment (Hamadeh et al., 2024d). Acceptance and adherence to interventions becomes greater when nurses understand the value of interventions and feel confident in their knowledge and skills (Durlak, 2017). Self-regulation, supported by access to information, resources, and organisational support, underpins autonomy and effective practice (Gottlieb et al., 2021; Messineoet al., 2019).

After highlighting contextual features conducive to successful implementation across organisational levels, we now discuss implications for stakeholders within these structures.

## 4 Implications

**Fig. 2:**
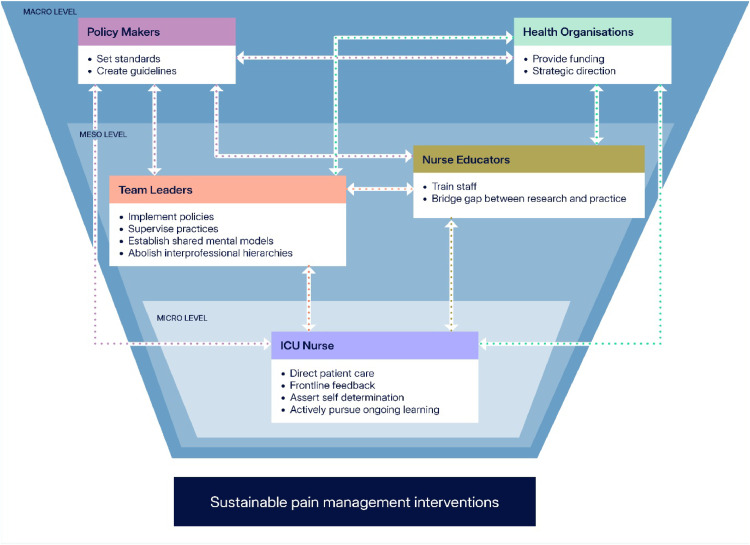
Interrelationships between stakeholders and sustainable pain management interventions at the different levels of the health organisation social system.

The Society of Critical Care Medicine advocates for an assessment-driven, protocol-based, stepwise approach to pain and sedation management (Devlin et al., 2018). Researchers have found that both untreated pain and opioid overuse in the intensive care setting are associated with chronic pain post-discharge and reinforces perceptions of pain as an adverse consequence of intensive care admission (Baumbach et al., 2016; Devine et al., 2019; Nordness et al., 2021). Physiologically, pain can destabilise metabolic and haemodynamic states through sustained adrenergic stimulation, leading to tissue injury and inflammatory responses (Burton et al., 2016). Pain also contributes to intensive care delirium, which has long-term cognitive implication (Leong et al., 2025). Despite numerous proposed interventions to enhance pain management, success varies across settings(Elliott et al., 2013). We have underscored the importance of context-sensitive implementation, shaped by stakeholder reasoning and resource availability. Empowering stakeholders, including policymakers, administrators, team leaders, nurses, and educators, promotes autonomy and confidence, which are essential for sustainable pain management practices. In Fig. 2, the interrelationships between the different stakeholders and sustainable pain management interventions are showcased at the different levels of the social system.

### 4.1 Implications for policy makers

Integrating evidence into healthcare interventions is essential for improving outcomes and is both achievable and necessary(Fontaine et al., 2024). Current pain assessment practices often fail to reflect best evidence, underscoring the need for stronger alignment between research and clinical implementation (Hamadeh et al., 2024a). Policymakers may be able to address this gap by conducting rigorous research prior to intervention design, identifying factors that enhance implementation success, and collaborating with diverse stakeholders. A comprehensive gap analysis prior to implementation is critical for identifying service delivery challenges and ensuring sustainability (Bierbaum et al., 2025). Such analysis also strengthens stakeholder buy-in by aligning interventions with recognised needs.

Effective implementation is a shared responsibility among policymakers, administrators, clinicians, and educators (Durlak, 2017). Stakeholders value inclusion in decision-making, which enhances motivation, empowerment, and commitment to improve practices (Hamadeh et al., 2024d). Collaborative approaches from inception to implementation foster ownership and drive success (Hamadeh et al., 2024d). Motivation to adopt interventions is influenced by perceived relevance, clinical utility, and adaptability (Durlak, 2017). Interventions gain support when they are comprehensive, accessible, user-friendly, and offer therapeutic alternatives (Hamadeh et al., 2024d). Preparatory education, regular evaluation, and clear performance benchmarks further enhance acceptability and adherence, promoting accountability and shared commitment to quality care (Hamadeh et al., 2024b).

### 4.2 Implications for health organisations

Healthcare organisations may be able to strengthen intervention buy-in by implementing actionable strategies supported by resources, leadership, and organisational readiness. These strategies should be context-specific, informed by stakeholder feedback, and continuously evaluated (Hamadeh et al., 2024d). Core approaches include building awareness through education, instituting quality assurance, prioritising pain management to improve outcomes, developing shared mental models, and addressing barriers to implementation (Hamadeh et al., 2024d).

Employing valid, evidence-based interventions fosters confidence in clinical decisions and reduces frustration caused by practice variability (Deldar et al., 2018; Mistraletti et al., 2017). Education and training are critical for shifting attitudes and overcoming biases toward pain management (Deldar et al., 2018; Radtke et al., 2012). Governance structures that incorporate performance evaluation and audits, coupled with constructive feedback, promote accountability and reflective practice without punitive connotations (Crawford et al., 2016; Yan et al., 2019).

Providing equitable education across interprofessional teams builds trust, dismantles hierarchical barriers, and enhances collaborative decision-making (Yan et al., 2019). Shared mental models improve communication, reduce adverse events, and standardise care (Brown et al., 2017; Riley et al., 2017). Resource allocation to manage workload and ensure adequate skill mix signals organisational support, mitigating such common barriers as time constraints and fostering empowerment (Alotni et al., 2023; Kydonaki et al., 2019).

Integrating pain management with related care domains, such as delirium, sleep, and agitation, through bundled approaches can standardise practice and improve outcomes (Balas et al., 2019; Ely, 2017). Proactive identification and mitigation of barriers, combined with ongoing evaluation, ensure smooth, sustainable, and cost-effective implementation (Penuela et al., 2019). Finally, recognising and supporting educators who lead training initiatives reinforces their critical role and strengthens the overall implementation process (Doyle et al., 2021).

### 4.3 Implications for intensive care team leaders

Intensive care team leaders play a pivotal role in fostering collaborative relationships, validating nurses’ contributions, and supporting their autonomy in decision-making (Hamadeh et al., 2024b, 2024d). Promoting shared mental models, adapting care priorities, and facilitating access to learning opportunities can strengthen clinical skills, expand knowledge, and encourage self-directed approaches to pain management (Hamadeh et al., 2024a). When leaders cultivate interprofessional collaboration to implement evidence-based solutions for undertreated pain, adherence to pain interventions improves (Hamadeh et al., 2024d). Leadership that fosters trust and shared accountability empowers staff to participate meaningfully in decision-making, enhancing inclusivity and reducing the disempowerment often associated with hierarchical structures (Batiha, 2014; Devlin et al., 2020; Tsang et al., 2019).Traditional dominance of physicians in decision-making reinforces professional hierarchies, limits collaboration, and constrains nurses’ scope of practice (Lokatt et al., 2019). Historically, medical authority has been shaped by the emphasis on specialised knowledge, often sidelining contributions from other professionals (Currie et al., 2012; Sanders & Harrison, 2008; von Knorring et al., 2016). When nurses’ input is overlooked, their role is reduced to task execution, undermining integrated care. Addressing these dynamics by team leaders is essential. By valuing nurses’ clinical judgment and promoting inclusive practices, team leaders can foster shared ownership of care decisions, reduce fear of blame, and strengthen professional autonomy (Kydonaki et al., 2019; Martínez et al., 2017).

Successful implementation of clinical interventions requires recognising that stakeholder support varies, particularly during early stages (Arnahoutova et al., 2024). While team members may acknowledge the value of change, commitment levels differ. High-performing teams are those supported by leaders who cultivate engagement and discretionary effort (Hamadeh et al., 2024d). In the intensive care settings, leadership that ensures access to learning, provides ongoing support, and encourages practical application of knowledge is critical for sustaining pain management interventions (Hamadeh et al., 2024d). Such practices empower nurses, foster enthusiasm for professional development, and promote a culture of continuous improvement (Latimer et al., 2010).

Moral distress, or psychological discomfort arising from inability to act in accordance with ethical beliefs (Choe et al., 2015), is prevalent in critical care, where rapid clinical changes and shifting treatment goals are common (Jakimowicz et al., 2017). Ethical tensions may stem from unresolved interprofessional disagreements or conflicting team priorities (Edelstein et al., 2009), team leaders can mitigate moral distress by validating nursing roles.

Prioritising pain assessment as a key performance indicator and emphasising its importance during inter and intraprofessional rounds can significantly influence intervention success (Hamadeh et al., 2024a). This is particularly relevant where financial incentives align with performance metrics, reinforcing accountability and continuous quality improvement(Deldar et al., 2018). Ineffective pain management is associated with prolonged hospital stays (Soares Pinheiro et al., 2020), a recognised indicator of healthcare quality (Chrusch & Martin, 2016). Intensive care leaders are therefore critical in disseminating information, building capacity, and acknowledging contributions to best practice.

### 4.4 Implications for nurses

Effective implementation of pain management interventions depends on nurses’ commitment to learning, ethical awareness, and confidence in autonomous decision-making (Hamadeh et al., 2024d). Reflective practice enables nurses to identify knowledge gaps and seek evidence-based resources, enhancing expertise and patient outcomes (Hamadeh et al., 2024b). Recognising these gaps promotes proactive engagement with interventions, fostering confidence and effectiveness of implementation (Hamadeh et al., 2024b). This highlights the need for a culture of continuous learning within healthcare settings (Hamadeh et al., 2024d).

Nurses’ attitudes also shape implementation. When nurses value pain assessment, documentation, and interprofessional communication, adherence improves (Hamadeh et al., 2024d). However, added time pressures may cause frustration (Payen et al., 2007). Nurses who succeed in adopting new interventions typically demonstrate self-efficacy, acquire skills through education, and feel supported throughout the process (Durlak, 2017). Conversely, poor knowledge undermines confidence and may lead to resistance, resulting in inconsistent application (Hamadeh et al., 2024d).

Professional autonomy is threatened by heavy workloads, unclear decision-making, and lack of recognition (Traynor et al., 2010). If interventions are perceived as complex or impractical, nurses may question their validity and importance (Deldar et al., 2018; Raurell-Torredà et al., 2019). Adequate support is therefore essential to build preparedness.

While clinical experience remains vital, reliance on it alone can hinder standardisation. Evidence-based interventions offer opportunities to improve consistency without compromising patient-centred care. Nurses must balance preserving autonomy with embracing practices that enhance quality and safety (Hamadeh et al., 2024d; Traynor et al., 2010).

**4.5 Implications for educators**

Ongoing, evidence-based educational programmes are essential for raising awareness, building capacity, and boosting confidence among nurses (Sedighie et al., 2020). Inadequate knowledge limits the effective use of pain assessment tools (Deldar et al., 2018) and impedes pain management (Schreiber et al., 2014). Accessible education and continuous professional development, supported by workplace structures that enable efficient practice, are monumental (Gottlieb et al., 2021). Educators can enhance intervention adoption by promoting awareness, delivering evidence-based and inclusive education, and collaborating with team members to ensure consistent, ongoing training.

Integrating training within interprofessional teams through varied, innovative learning sessions enhances knowledge acquisition, strengthens assessment skills, and fosters self-efficacy (Hamadeh et al., 2024d); (Deldar et al., 2018; Mistraletti et al., 2017). Targeted education for nurse leaders can empower them to champion interventions and demonstrate adaptive leadership (Stone et al., 2020). Previous studies emphasised the value of analogous training for nurses and doctors to ensure consistent pain management (Chanques et al., 2007; Gélinas, 2010). Educators could implement team-wide training to promote uniform assessment, scoring, and documentation practices within the interprofessional team. Such approach helps bridge hierarchical gaps and foster equality within interprofessional teams (Hamadeh et al., 2024d).However, intensive care environments pose challenges to education delivery, including diverse educational backgrounds, time constraints, clinical uncertainty, and frequent interruptions (Carlos et al., 2016; Piquette et al., 2015; Tainter et al., 2017). To overcome these barriers, educators could model behaviours that create an inclusive and engaging learning experience, such as demonstrating enthusiasm for teaching, showing empathy, explaining clinical reasoning clearly, treating staff respectfully, and maintaining a safe learning environment (Doyle et al., 2021; Santhosh et al., 2017).

Empowering a clinical nurse specialist or nurse champion who is embedded within the team can further strengthen education and engagement during implementation (Hamadeh et al., 2024d). Including pain management training in induction programmes for new staff also promotes consistency and shared understanding from the outset (Hamadeh et al., 2024d).

## 5 Recommendations

Building on previous insights, we propose the following recommendations to optimise pain management for sedated and ventilated patients. Collaborative engagement from all stakeholders may be essential to achieve this goal and improve outcomes across organisational levels. Table 2 provides a summary of these recommendations.

**Table 2:** Summary of the recommendations involving different stakeholders, relating to effective implementation of pain management interventions in intensive care setting.

Stakeholder	Recommendations
Policy Makers	Design evidence-based, user-friendly pain management interventions. Translate guidelines into practice using implementation science. Use research findings to inform conditions for sustainable implementation.
Health Organisations	Invest in infrastructure and resources. Identify and address barriers to implementation. Foster stakeholder relationships through communication. Provide ongoing education and quality assurance. Offer constructive feedback in a supportive environment.
Unit Leaders	Promote interprofessional collaboration and shared mental models. Challenge professional hierarchies and support nurses’ autonomy. Create safe spaces for discussion and innovation. Advocate for nurses’ roles in intervention development. Provide coaching, feedback, and recognition systems. Address team tensions to enhance performance.
Nurses	Update knowledge and skills in pain management. Understand consequences of poor pain assessment. Embrace change and assert professional identity. Accept and act on constructive feedback.
Educators	Update pain management evidence. Facilitate inclusive education across shifts and employment types. Engage champions (e.g., clinical nurse specialists) for bedside consistency. Integrate advanced pain management education into nursing curricula. Ensure induction programs include pain management skills.

## 6 Strengths and limitations

We have synthesised evidence from a realist evaluation study, emphasising the critical role of contextual features (sociocultural, organisational, professional, and individual) in shaping the successful implementation of pain management interventions in intensive care settings. Given the limited research in this domain, these insights are particularly valuable for guiding practice.

Realist evaluations aim to uncover context–mechanism–outcome configurations; however, real-world contexts are often highly complex and dynamic. Capturing all relevant contextual variables can be challenging, sometimes resulting in incomplete or oversimplified explanations. While realist evaluation offers rich insights into “what works, for whom, and under what circumstances,” its transferability to other settings may be limited (Pawson & Tilley, 1997). Furthermore, program theories were developed and refined through interpretive processes, which can introduce researcher bias if not carefully managed. To mitigate this risk, we employed an iterative process of retroduction and ensured transparency throughout (Hamadeh et al., 2024d).

Future researchers should examine how the refined theories apply to different stakeholder configurations (e.g., physicians, allied health professionals, and hospital administrators) and explore their responses to implementation. Additionally, studies should assess the sustainability of interventions and prioritise comprehensive, consistent reporting to strengthen the evidence base and inform future implementation efforts.

## 7 Conclusion

Inconsistent pain management not only compromises patient outcomes but may also undermine clinician confidence and team cohesion. Embedding evidence-based interventions within supportive contexts can improve psychological, physiological, and social wellbeing for patients, while also enhancing professional engagement. A system-wide approach, spanning macro (policy and governance), meso (organisational and team), and micro (individual clinician) levels, may foster a culture of trust, autonomy, and accountability. For practitioners, this means prioritising collaborative structures, reflective practice, and continuous feedback mechanisms to promote sustainable and effective pain management strategies in the intensive care setting.

## Declaration of generative AI and AI-assisted technologies in the manuscript preparation process

During the preparation of this work the authors (Samira Hamadeh and Loretta Garvey) used CoPilot in order to ensure concise and clear expression throughout the manuscript. After using this tool, the authors reviewed and edited the content as needed and take full responsibility for the content of the published article.

## Funding

We did not receive any specific grant from funding agencies in the public, commercial, or not-for-profit sectors.

## CRediT authorship contribution statement

**Samira Hamadeh:** Writing – review & editing, Writing – original draft, Project administration, Methodology, Investigation, Formal analysis, Conceptualization. **Loretta Garvey:** Writing – review & editing, Supervision, Conceptualization.

## Declaration of competing interest

The authors declare that they have no known competing financial interests or personal relationships that could have appeared to influence the work reported in this paper.
